# *Lomentospora prolificans* infective endocarditis and cerebral mycotic aneurysm

**DOI:** 10.1016/j.mmcr.2026.100771

**Published:** 2026-02-10

**Authors:** Robert Hand, J. Owen Robinson, Paul Cannell, Robert Larbalestier, Thomas Gliddon, Peter Boan

**Affiliations:** a Infectious Diseases Department, Fiona Stanley Hospital, 11 Robin Warren Ave, Murdoch, Perth, 6150, Australia; b Microbiology Department, Fiona Stanley Hospital, PathWest Laboratory Medicine WA, 11 Robin Warren Ave, Murdoch, Perth, 6150, Australia; c Infectious Diseases Department, Royal Perth Hospital, Victoria Square, Perth, 6000, Australia; d Haematology Department, Fiona Stanley Hospital, 11 Robin Warren Ave, Murdoch, Perth, 6150, Australia; e Cardiothoracic Surgery Department, Fiona Stanley Hospital, 11 Robin Warren Ave, Murdoch, Perth, 6150, Australia; f Infectious Diseases Department, Sir Charles Gairdner Hospital, Hospital Ave, Nedlands, Perth, 6009, Australia

**Keywords:** Lomentospora, Olorofim, Endocarditis, Mycotic aneurysm, Transplant

## Abstract

Disseminated *Lomentospora prolificans* infection typically occurs in immunocompromised hosts and is almost universally fatal. We describe a case of *L. prolificans* native valve infective endocarditis with cerebral mycotic aneurysm after recovery from allogeneic stem cell transplant. The case is notable for minimal immunosuppression at diagnosis, and pathological evidence of infection at sites which looked normal at surgery or by imaging. With early surgery and olorofim the patient survived to 76 days after diagnosis.

## 1 Introduction

*Lomentospora prolificans* (*L. prolificans*) is a dematiaceous mould and an emerging opportunistic pathogen. The most common presentations relate to traumatic inoculation or disseminated disease in the immunocompromised host [1]. Cases are rare, with a review in 2019 identifying 56 cases of invasive *L. prolificans* since 2000 [2]. A review of Australian cases identified 37 cases of *L. prolificans* infection between 2005 and 2021 [1].

In this case series risk factors for *L. prolificans* infection included haematological malignancy, haematopoietic transplant and prolonged neutropenia. Infection was disseminated in 73%. The 30-day mortality rate with disseminated disease was very high at 70.3%, and median survival was only 6 days (range 0-433 days) [1].

Combination antifungal therapy of voriconazole and terbinafine with adjunctive surgery are suggested by guidelines [[3], [4], [5]], with an evolving role of olorofim as a more active in-vitro agent [6]. Olorofim is a novel antifungal which inhibits the pyrimidine biosynthesis enzyme dihydroorotate dehydrogenase [6].

We present an illustrative case of *L. prolificans* infection causing infective endocarditis and cerebral mycotic aneurysm. The case was unusual for prolonged survival to 76 days after diagnosis, providing a number of insights arising from the clinical management. Early mitral valve surgery and olorofim-based antifungal therapy are postulated to have contributed to longer than predicted survival. Nevertheless, infection diagnosed by histopathology and culture of operative specimens had advanced further than could be appreciated macroscopically or by multimodal imaging. In desperation with the failure of other therapy, we utilised intrathecal and high dose systemic echinocandin and discuss aspects of this approach in relation to the published literature. The patient gave written signed consent for publication of the case.

## 2 Case presentation

### 2.1 Background

A woman in her 30s was diagnosed with acute myeloid leukemia (AML) in 2021. Her third chemotherapy cycle in June 2022 was complicated by a nodular lung lesion on computed tomography (CT) chest and concurrent clinical syndrome of meningitis. Bronchoalveolar lavage and CT guided core lung biopsy 10 days after the substitution of posaconazole prophylaxis for liposomal amphotericin B (LAmB) 3 mg/kg daily and voriconazole did not yield evidence of fungal infection. Cerebrospinal fluid (CSF) demonstrated elevated white cell count (WCC) of 240 x 10^9^/L, (normal range [NR] <5 x10^9^/L), 70% neutrophils, protein 0.65 g/L (NR 0.15-0.45g/L), glucose 2.8 mmol/L (NR 2.8-4.4 mmol/L). CSF fungal and bacterial culture, viral and bacterial polymerase chain reaction (PCR), cryptococcal antigen were all negative. She received 10 days of meropenem. LAmB and voriconazole were switched after 3 weeks back to posaconazole due to liver derangement, which resolved and trough levels were maintained at 0.9-1.2 mg/L. Lung nodules were reduced after 6 weeks and she proceeded to a matched unrelated donor haematopoietic cell transplant in September 2022 with pre- and post-transplant posaconazole. CT chest Jan 3, 2023 (day 111 post-transplant) showed a linear opacity in the right upper lobe 8 × 3 mm unchanged from August 2022. Posaconazole was ceased Jan 18, 2023. At this time, her WCC was 3.21x10^9^/L (NR 4-11x10^9^/L) neutrophils 2.05 x10^9^ (NR 2-7.5x x10^9^/L) lymphocytes 0.72 x10^9^/L (NR 1.2- 4.0 x10^9^/L) CD4^+^ lymphocytes 102 x10^6^/L (NR 457-1498 x10^6^/L), CD8^+^ lymphocytes 450 x 10^9^/L (NR 205-1013 x10^6^/L, CD19^+^ lymphocytes 62 x 10^6^/L (NR 93-480 x10^6^/L. Total IgG, IgM, IgA were within normal limits. She had no graft-versus-host-disease (GVHD) and was on ciclosporin (50 mg bd).

### 2.2 Diagnosis

**Fig. 1:**
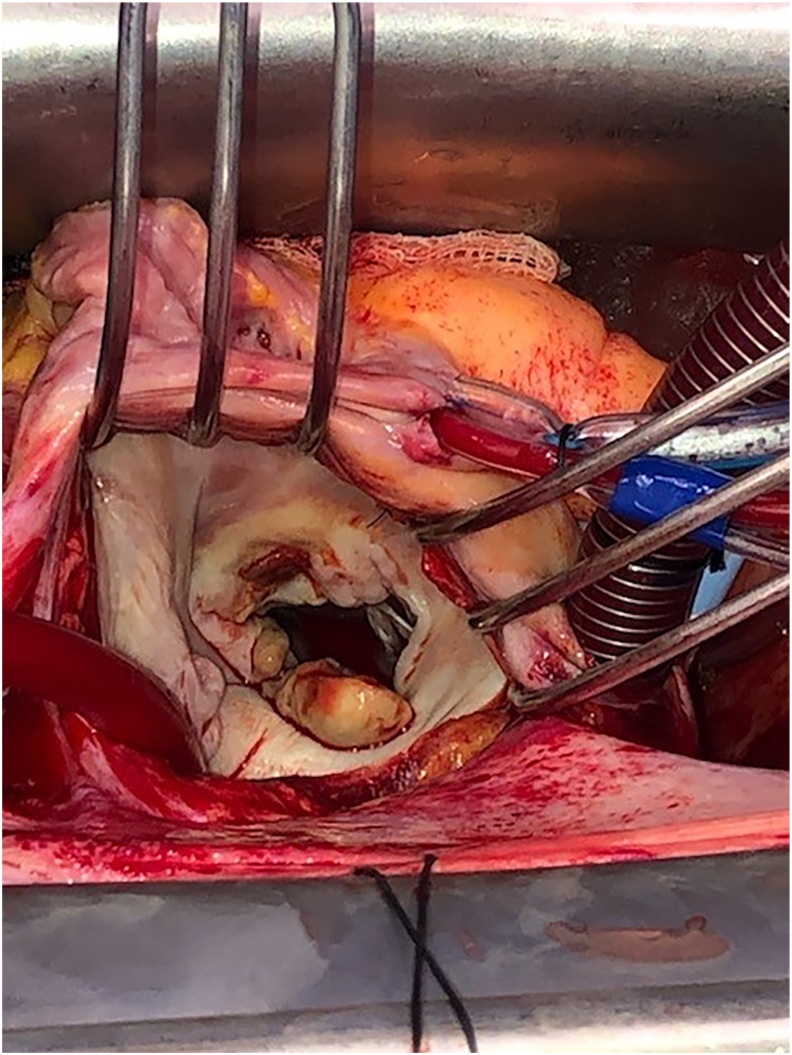
Large vegetation on the native mitral valve.

The main diagnostic and treatment events are listed in Table 1. Fever began on Jan 31, 2023 and blood cultures grew *L. prolificans* (Day 0), identified by morphological features, matrix-assisted laser desorption/ionisation time of flight mass spectrometry (MALDI Biotyper®, Bruker, MA, USA) score >2.0, and in-house internal transcribed spacer 1 and 2 gene sequencing as previously described [7]. Minimum inhibitory concentrations (MICs) by Sensititre YeastOne SYO10 microbroth dilution were: voriconazole >8 mg/L, posaconazole >8 mg/L, itraconazole >16 mg/L, isavuconazole >8 mg/L, amphotericin B > 8 mg/L. Minimum effective concentrations (MECs) were: anidulafungin 0.5 mg/L, micafungin 2 mg/L. Olorofim MIC was 0.125 mg/L. Examination demonstrated a mitral regurgitation murmur. Contrast CT head/chest/abdomen showed no significant abnormalities. Trans-oesophageal echocardiogram (TOE) showed a large 1.7 cm mitral valve (MV) vegetation with regurgitation and she proceeded to mechanical MV replacement D+9 (Fig. 1.). Valve tissue grew *L. prolificans* and histopathology showed fungal elements present at the surgical margin. She had one 30 second episode of altered vision in one eye prior to surgery and a small pale retinal lesion with subretinal haemorrhage on examination. A Gallium-67 citrate scan at D+15 was non contributary, as was a D+20 18-FDG positron emission tomography (PET) which demonstrated only post-surgical changes. Magnetic resonance imaging (MRI)/magnetic resonance angiogram (MRA) of the brain with contrast D+20 showed 3 small watershed contrast enhancing, diffusion restricting lesions consistent with fungal septic emboli. There was no evidence of mycotic aneurysms on PET or MRA.

**Table 1:** Timeline of main diagnostic and treatment events.

Day from diagnosis	Diagnostic event	Treatment event
−139	-	Allogeneic stem cell transplant for acute myeloid leukemia
0	Blood cultures taken grew *L. prolificans*	-
+4	-	Voriconazole plus terbinafine
+7	Evidence of mitral valve endocarditis, retinal lesions, cerebral septic emboli	Olorofim added to voriconazole and terbinafine
+9	-	Mechanical mitral valve replacement
+10 and + 17	-	Intravitreal voriconazole
+23	Eye examination stable	Olorofim 120 mg daily single agent. Voriconazole and terbinafine ceased
+37	Blood culture again grew *L. prolificans.* Echocardiographic evidence of relapsed endocarditis	Olorofim dose increased to 150 mg twice daily
+52	-	Redo mitral valve replacement. Intravenous anidulafungin 200-400 mg daily added to olorofim
+60	Subarachnoid haemorrhage from basilar artery mycotic aneurysm	Externalventricular drain, endovascular coiling of aneurysm
+70	New basilar artery aneurysm	Further endovascular coiling. Intrathecal caspofungin
+76	Death

### 2.3 Treatment

Antifungal therapy consisted of voriconazole and terbinafine from D+4, and olorofim was added D+7 (150 mg bd on day 1 loading dose, then 90 mg bd). Although voriconazole and olorofim are possibly antagonistic in vitro [8], voriconazole was continued at this time due to its high-level penetration into the vitreous and brain. Intravitreous voriconazole was administered D+10 and D+17. With stability in examination findings in the eye, olorofim was continued as a single agent from D+23, increasing from 90mg bd to 120 mg bd to account for cessation of voriconazole. Blood cultures taken D+11 through D+35 were negative.

**Fig. 2:**
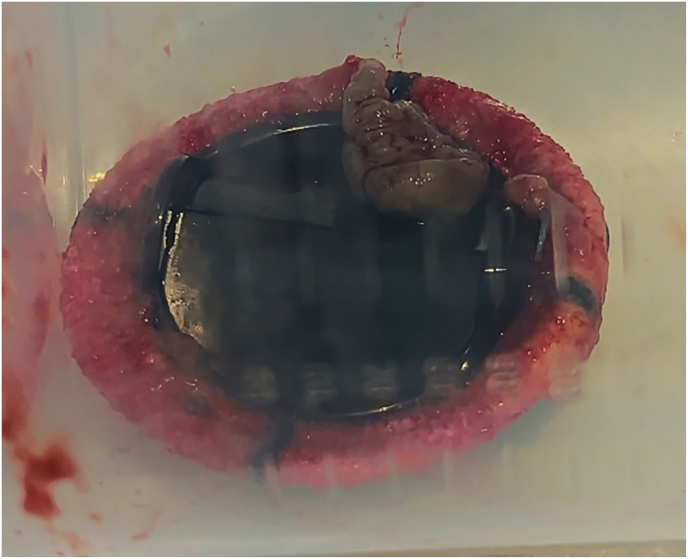
Explanted prosthetic valve demonstrating a large vegetation at the sewing ring.

Blood culture D+37 grew *L. prolificans* so olorofim was increased to the maximum dose of 150 mg twice daily. MRI brain with contrast D+45 showed resolution of prior small lesions. TOE showed a large highly mobile echodensity attached to the sewing ring of the prosthetic MV. She proceeded to redo surgery D+52 where the sternal bone was macroscopically abnormal, microscopy and culture positive for *L. prolificans*. The pericardium was unremarkable. A large margin around the prosthetic valve (Fig. 2.) was excised. Perivalvular biopsies from macroscopically normal areas grew *L. prolificans*. Adjunctive antifungals were reconsidered. Voriconazole was not added for possible antagonism with olorofim. We were also concerned that if voriconazole caused liver dysfunction this might have necessitated reduction or cessation of olorofim, the most active in-vitro agent. Terbinafine is mainly active through synergy with voriconazole so this was not utilised. Anidulafungin was added at a dose of 400 mg once daily as the patient's isolate had a MEC at the lower end of usual for this organism (0.5 mg/L). Heart block occurred after high dose anidulafungin. Anidulafungin was reduced to 200 mg daily, a pacemaker was inserted, after which the dose was increased to 400 mg daily ongoing. Blood culture D+57 was negative.

### 2.4 Progress and outcome

**Fig. 3:**
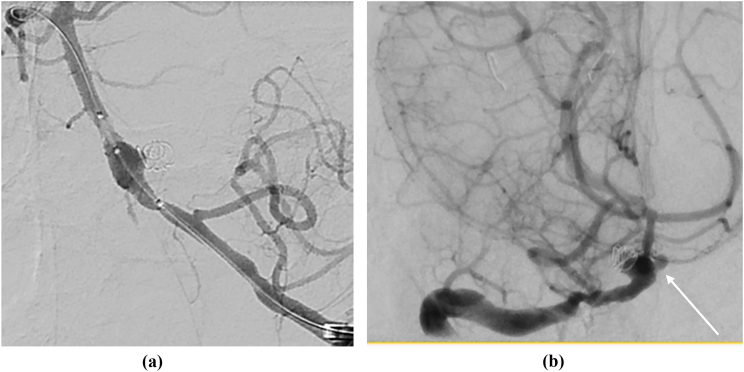
a/3bDigital subtraction angiography demonstrating 5mm coiled basilar mycotic aneurysm (Fig. 3a) at D+60 and interval development of further 2.3mm mycotic aneurysm at D+70 (Fig. 3b, white arrow).

On D+60, she developed headache, reduction in consciousness, and CT head showed grade 4 subarachnoid haemorrhage with early hydrocephalus. An external ventricular drain was inserted. Cerebral angiography demonstrated a 4mm mycotic right basilar artery aneurysm, which was endovascularly coiled (Fig. 3.). Despite our prior rationale, with progressive disease voriconazole and terbinafine were added to anidulafungin and olorofim (dose reduced to 120 mg bd). She had rising intracranial pressure D+70, when a new left 2.3 mm basilar artery mycotic aneurysm and progressive right basilar aneurysm were both endovascularly coiled. Intrathecal caspofungin was added to therapy (5 mg in 5 mL of normal saline daily with 30-60 min clamp time) given prior case reports of its use [9,10]. CT chest/abdomen/pelvis showed new splenic and kidney infarcts and blood culture grew *L*. *prolificans* from D+67 onwards. CSF and urine also grew *L. prolificans*. Echocardiogram D+63 did not show an obvious vegetation. She had further intracranial haemorrhage, progressive cerebellar tonsillar herniation, and died D+76.

## 3 Discussion

This complex case of *L. prolificans* infection instructed us in relation to patient survival, antifungal therapy, and organism tissue invasion and predilection.

The patient survived to D+76, where median survival in disseminated disease is six days (range 0-433 days) [1]. Immune function was better in our patient than many who develop disseminated *L. prolificans* infection in that she did not have GVHD or neutropenia; was on low dose ciclosporin; lymphocyte count and immunoglobulin levels were recovering. Early aggressive surgical source control improves outcome and proceeding to mitral valve resection D+9 achieved early debulking [3]. Early access to olorofim was likely also important, as an antifungal agent with demonstrated in vitro activity against *L. prolificans* [3]. Clinical trial outcome data with olorofim for lomentosporiosis showed stable disease in 73.1% and mortality of 11.5% at Day 84 of treatment, however only 31% had disseminated disease and 4% CNS disease [6].

Echinocandins cannot be recommended for *L. prolificans* treatment as they usually test with elevated MECs in the range of 2-16 mg/L [11], and we do not have clinical evidence of efficacy. We used them intravenously and intrathecally in this case out of desperation, when surgery and olorofim failed to control the infection. We noted echinocandins may demonstrate synergy with other agents against *L. prolificans* [12], and the isolate from our patient tested with lower MECs of 0.5 mg/L for anidulafungin and 2 mg/L for micafungin. We utilised high dose anidulafungin (the echinocandin utilised at our site) at 400 mg once daily. We noted high doses of anidulafungin 300 mg every 72 hours [13], and micafungin 8mg/kg daily up to 900 mg have been utilised without obvious adverse effects [14]. Arrythmias and cardiac toxicity with echinocandins have been reported in animal studies and case reports [[15], [16], [17]], although we are not sure if heart block was an adverse effect of high dose anidulafungin in this case, as there were other potential contributors such as intracardiac infection and cardiac surgery.

Echinocandins have low central nervous system (CNS) penetration. Intraventricular caspofungin has been used at 5 mg/day for 14 days to treat CNS *Scedosporium apiospermum* infection with fungal clearance [10]. In another report of *Candida auris* CNS infection intraventricular caspofungin was given at 10 mg/day [9]. No direct side effects of administration were reported in these cases. We only had opportunity to utilise one intraventricular caspofungin dose before neurological decline from progressive infection, so cannot comment on efficacy or toxicity.

Infection was present further than we could appreciate. The first surgery excised the infected MV to macroscopically clean tissue, yet histopathology showed fungal elements to the margin of the excision and there proved to be local recurrence. At the second surgery surveillance biopsies from macroscopically uninfected endocardium of the mitral annulus grew *L. prolificans*. Additionally, PET scan D+20 and CT chest D+43 demonstrated no significant peri-sternal abnormalities despite the presence of infection at surgery D+52. Operative frozen section may have a role in ensuring microscopically clear surgical margins.

We present an illustrative case of disseminated *L. prolificans* infection with infective endocarditis and cerebral mycotic aneurysm in a patient recovering from allogeneic stem cell transplant. With early MV surgery and olorofim she survived longer than predicted from previous studies. Infection had advanced further than we could appreciate macroscopically or by multimodal imaging.

## CRediT authorship contribution statement

**Robert Hand:** Writing – review & editing, Writing – original draft, Visualization, Validation, Methodology, Investigation, Formal analysis, Conceptualization. **J. Owen Robinson:** Writing – review & editing, Visualization, Supervision, Project administration, Investigation, Formal analysis, Conceptualization. **Paul Cannell:** Writing – review & editing, Visualization, Validation, Supervision, Project administration, Investigation, Conceptualization. **Robert Larbalestier:** Writing – review & editing, Visualization, Validation, Supervision, Project administration, Investigation, Conceptualization. **Thomas Gliddon:** Writing – review & editing, Supervision, Project administration, Investigation, Formal analysis, Conceptualization. **Peter Boan:** Writing – review & editing, Writing – original draft, Visualization, Validation, Supervision, Project administration, Methodology, Investigation, Formal analysis, Conceptualization.

## Conflict of interest

There are none.
